# Crop performance and soil fertility improvement using organic fertilizer produced from valorization of *Carica papaya* fruit peel

**DOI:** 10.1038/s41598-021-84206-9

**Published:** 2021-02-25

**Authors:** S. O. Dahunsi, S. Oranusi, V. E. Efeovbokhan, A. T. Adesulu-Dahunsi, J. O. Ogunwole

**Affiliations:** 1 Microbiology Programme, College of Agriculture, Engineering and Science, Bowen University, Iwo, Osun State Nigeria; 2 Department of Biological Sciences, Covenant University, Ota, Ogun State Nigeria; 3 Department of Chemical Engineering, Covenant University, Ota, Ogun State Nigeria; 4 Food Science and Technology Programme, College of Agriculture, Engineering and Science, Bowen University, Iwo, Osun State Nigeria; 5 Agriculture Programme, College of Agriculture, Engineering and Science, Bowen University, Iwo, Osun State Nigeria

**Keywords:** Biotechnology, Microbiology, Plant sciences

## Abstract

In recent times, research attention is focusing on harnessing agricultural wastes for the production of value-added products. In this study, the valorization of *Carica papaya* (Pawpaw) fruit peels was evaluated for the production of quality organic fertilizer via anaerobic digestion (AD) while the effects of the fertilizer on maize crop were also assessed. Pawpaw peel was first pretreated by thermo-alkaline methods before AD and analyses were carried out using standard methods. The resulting digestate was rich in nutrients and was dewatered to form solid organic fertilizer rich in microbes and soil nutrients. When applied to maize plants, organic fertilizer showed a better effect on plant traits than NPK 15–15–15 fertilizer and without fertilizer application. These were more pronounced at mid to high organic fertilizer applications (30-to-60-kg nitrogen/hectare (kg N/ha)) rate. Comparison between the values obtained from the field experiments reveals that the organic fertilizer showed better performance in all parameters such as the number of leaves, leaf area, plant height, stem girth, total shoot, and root biomass, and length of the root. However, the chemical fertilizer outperformed all the organic fertilizer applied rates in the average highest size of the corn ear by 1.4%. After harvesting, nutrient elements were found to have bioaccumulated in plant organs (leaves, stem, and root) with the highest values being 29.7 mg/L for nitrogen in the leaf and this value was reported from the experiment with 50 kg N/ha. For phosphorus and potassium, the highest concentrations of 7.05 and 8.4 mg/L were recorded in the plant’ stem of the experiment with 50 kg N/ha. All the treated soils recorded an increase in values of all nutrient elements over the control with the highest values recorded in the experiment with 60 kg N/ha. In soil with 60 kg N/ha, the nitrogen, phosphorus, and potassium increased by 28, 40, and 22% respectively over the chemical fertilizer applied experiment while different levels of increases were also recorded for all other macro and microelements in all the experiments. Thus, agricultural practices by using anaerobic digestates as organic fertilizers is a sustainable method to overcome the dependence on inorganic fertilizers high rate.

## Introduction

Climate change and its attendant issues have been a major subject of discussion between scientists and researchers over the last decade. The bulk of the contributions to climate change comes from fossil fuels and their derivatives which now call for a radical approach in protecting the environment for human and animal survival^1^. One major way to achieve this is by shifting from a fossil fuel economy to a bio-based one which includes the identification of suitable materials (Wastes, biomass, etc.) that are useful for renewable energy generation and their subsequent conversion to such^2^. Of these renewable energies is biogas which does not depend largely on the volatile weather conditions unlike others such as wind and solar power^3^. Biogas is usually produced during the Anaerobic Digestion (AD) of organic materials such as solid wastes streams, greenery biomass, crop residues and animal dung, municipal and domestic wastes^4,5^.

Besides these numerous advantages of the AD process is the production of digestate as a by-product of the process and they vary in their intrinsic characteristics depending on the nature and composition of the material used in their production^7^. There is, therefore, a need to implement a sustainable method for storage, disposal, and management of digestate to avoid challenges of handling, environmental contamination, and odor^8^. The most promising application of digestate is in the agricultural and horticultural sectors where they are often applied as soil conditioners and organic fertilizers due to their richness in nutrients and soil viable microorganisms^9,10^. Digestates are so used due to their ability to improve and modify soil structures^11^, while they also improve soil nutrient status and boost a load of beneficial microorganisms for special functions, especially in marginal or nutrient-depleted soils when applied as organic fertilizers^12^. In this regard, digestates have been seen to be potentially able to partially or wholly replace inorganic chemical fertilizers in agricultural practices especially in tropical countries most of which are facing depletion in soil nutrients, toxicity to soil microorganisms, inadequate soil aeration, soil water pollution, and eutrophication^13^. Similarly, digestates are potent to replace the widespread application of peat which has special properties making it important for large-scale application as a growth media in horticulture^14^. More importantly, peat is a slowly-renewable and finite resource and thus does not have sustainable management methods which further make its usage environmentally controversial^15,16^.

Considering the afore-mentioned, a veritable alternative to both inorganic fertilizer and peat is anaerobic digestate which has proven to be a slow-release fertilizer providing essential nutrients such as nitrogen, phosphorous, and potassium (NPK) besides other essential plant macronutrients required for growth, health, and wellbeing of crop plants without detrimental effect on the soil^17,18^. This also goes a long way to boost food production to cater for the teeming human population thereby attaining the sustainable development goals (SDGs) one, two, and thirteen which are eradication of poverty, zero hunger, and climate action respectively^19,20^. Digestates contains different concentrations of nutrients especially NPK^21^ and have therefore made their usage of immense benefit to agriculturists since these nutrients are naturally scarce especially phosphorus which is usually obtained from mining with huge costs and high energy expenditure besides the serious health hazards posed by its mining^22^. Dependence on these natural and limited sources of nutrients for crop plants, therefore, makes agriculture vulnerable and less economical in the long run. In most cropping systems, the use of inorganic fertilizers have been abused and besides the huge cost of procurement, shipping, transportation, and distribution logistics, they pose serious threats to the environment with their attendant tendencies to reduce the integrity of soil, cause degradation of the environment, poses risks to biodiversity, high contribution to an algal bloom, and their huge potentials to make soil heavy metals laden^16^. The situation can however be redeemed via the adoption and continuous use of organic preparations e.g., such as digestate organic fertilizer produced from renewable and locally available bioresources^6,23^.

*Carica papaya* (Papaya; Pawpaw in the local name) is a globally important and popular fruit presently with a total of 11.22 metric tons equivalent of 15.36% of the total production of tropical fruits^24^. About 60 countries are known for papaya production globally in which the bulk comes from developing nations in the tropics. Production of this fruit has grown considerably over the last two decades from a total of 7.25 metric tons in 2000 to 13.02 metric tons in 2017^25^. The five-leading papaya-producing nations are India, Brazil, Indonesia, Nigeria, and Mexico with 38.61, 17.5, 6.89, 6.79, and 6.18% respectively. Being the 4th leading papaya producing nation in the world, Nigeria witnesses massive production of this fruit annually, and its flesh/pulp is either consumed raw or used in various preparations. However, the huge peels accrued from papaya processing remain an environmental nuisance as solid wastes and serving as vehicles for transmission of life-threatening diseases since there are no sustainable management methods for this massive and year-round bioresource. This has created a huge knowledge gap in sustainably managing papaya fruit peels by converting them to value-added products while protecting environmental integrity. Though few previous studies have reported the production of organic fertilizer from pawpaw fruit peels, such studies were carried out using mixed fruit peels such as banana, pineapples, papaya, pomegranate, sweet lime, orange, etc. which resulted in some organic preparations that cannot be distinctly categorized based on the source of its raw materials^26,27^. This current study represents a novel and modern trend in the sole utilization of pawpaw fruit peels as a veritable and abundant resource for organic agriculture improvement. Therefore, this study aimed to evaluate the valorization of pawpaw fruit peels for the production of quality organic fertilizer. This is important to establish the peel as a profound bioresource in global organic agricultural development, reduce solid waste accumulation in the environment with its attendant public health menace as well as documenting a sustainable management method for pawpaw peel in the long run. The effects of the fertilizer on the performance of maize (*Zea mays*) as test plants will be assessed while the possibility of soil fertility improvement as a result of the organic fertilizer application will also be evaluated. It is hoped that these tests will help validate the potency of the produced fertilizer in the general wellbeing of the crop plant (Maize) and will engender its further usage on other crop plants besides increasing productivity and environmental protection in a circular economy.

## Materials and methods

### Sample collection and pretreatment

**Figure 1:**
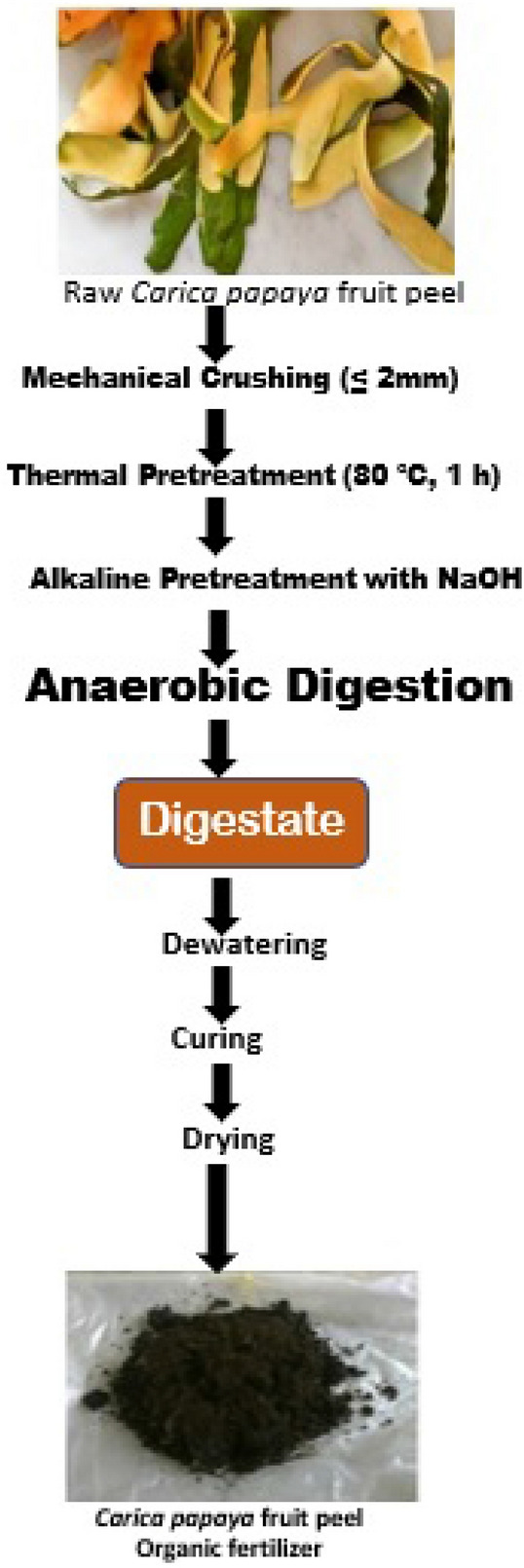
Pictorial representation of the organic fertilizer development process.

Papaya fruit peel (Fig. 1) was obtained from Landmark University Farms and staff quarters while the microbial inoculum i.e., cattle rumen content was collected into a sterile container from an abattoir. Due to its high content of lignin and cellulose, papaya fruit peel needed to be pretreated to make it easily biodegradable and to avoid the usual rate-limiting occurrence especially during the hydrolysis stage of digestion. In achieving this, three different pretreatment methods i.e. mechanical, thermal, and alkaline (NaOH) were applied as described in previous studies^28–36^. The peels were initially crushed into sizes of ≤ 20 mm using a hammer mill and this was followed with 80 °C thermal treatment in a water bath (CLIFTON, 88,579, Nickel-Electro Ltd., England). The temperature was chosen base on the results of previous works which showed it as the optimal for a suitable lignocellulosic pretreatment^37,38^. The procedure for alkaline pretreatment was carried out using 3 g NaOH/100 g TS at 55° C for 24 h^39^. The choice of NaOH was a result of its earlier performance as a suitable alkali for lignocellulosic biomass pretreatment^40^.

### Anaerobic digestion

The slurry was prepared from the pretreated biomass and water and it was anaerobically treated in controlled batch anaerobic reactors using. The collected bovine rumen fluid was activated by a sieve to remove unwanted biomass and dirt, poured into a clean container, and flushed the headspace with hydrogen gas before incubation for 5 to 7 days. This activated inoculum was used to seed the reactors and they were turned on for digestion to produce biogas over a retention period of 30 days^28–34^. The batch anaerobic reactor (EDIBON, England) with twin digestion chambers each of 5-L capacity was employed. The reactors were computer-controlled with internal probes to measure the pH and temperature. Before digestion, a sample of the prepared slurry was taken for physical, chemical, and microbiological analyses. Also, during the digestion, samples were taken weekly for the same analyses. The produced biogas was collected via the water displacement unit which is a component of the automated reactors.

### Organic fertilizer development procedures

At the expiration of the AD, the digestates were carefully removed from the reactors, and samples were taken for analysis before digestates have been poured into sterile sacks to drain. Curing of the dewatered digestate was carried out for 20 days using sterile bags and the resulting solid preparation was stored in the dry forms^41,42^ before physicochemical and nutrient analysis as well as application in a field experiment.

### Analytical procedures

There is a need to adequately characterize substrates for AD to determine their intrinsic components which further enhance their performance during production^43^. Physical properties (pH and Temperature) of the fermenting materials, inoculum, digestates, and solid organic fertilizers were determined. After this, chemical analyses of the same materials were carried out to quantify their elemental and nutrients compositions. An inductively coupled plasma mass spectrometry was used to determine the total carbon, nitrogen, phosphorus, potassium, phosphate, sulfate, calcium, magnesium, manganese, iron, zinc, aluminum, and copper^35^. To determine the Chemical Oxygen Demand (COD) of samples, the method of the American Public Health Association (APHA) for analyzing water and wastewaters^44^ was used. The values of Total solids (TS), volatile solids (VS), ash, and moisture contents were determined using the Finnish Standard Association SFS 3008 protocol^45^.

### Microbiological analyses

#### Characterization and enumeration of aerobic and anaerobic bacteria and fungi

The standard method for total aerobic plate count was adopted in analyzing the aerobic bacteria in the fermenting material; inoculum, digestate, and solid organic fertilizer in this study employing nutrient agar, eosin methylene blue (EMB) agar, peptone water, and MacConkey agar. All samples were collected aseptically and in triplicates while presumptive isolates were characterized by phenotypic methods and the probable ones further identified using appropriate rapid API kits (BioMerieux, France) as previously reported^28–33^.

To characterize the anaerobic bacteria, samples were initially cultured on two enriched media (Reinforced Clostridia medium and blood agar) in anaerobic chambers at 37º C between 5 and 7 days. This was meant to detect members of the *Clostridia* and other facultative anaerobes in the samples. After this, Brain Heart Infusion agar was employed in fully growing developed colonies followed by counting and recording^46^. The phenotypic features of the presumptive isolates were determined after which appropriate rapid API kits were used for their final confirmation^47^. For fungal evaluation, samples were cultured on Potato dextrose agar and grown for 5–7 days before identification by hypha and spore morphology and those of the fruiting bodies^48^.

#### Enumeration of methanogen (archaea)

In some previous investigations on the characterization and identification of methanogens, a mineral-rich basal medium was compounded, utilized, and was found very efficient^28–34^. The medium was employed in characterizing the methanogens in the sample of fermenting material, inoculum, digestate, and solid organic fertilizer in this study using the same protocol.

### Phyto-assessment with Maize (*Zea mays*) using produced organic fertilizer

The organic fertilizer was applied to maize plants to ascertain their nutrient level especially with regards to their nitrogen content since this element plays a vital role in plant growth plant and during protein formation. The experiments were carried out during the 2015 and 2016 cropping seasons at the Teaching and Research Farm of Landmark University, Omu-Aran, Nigeria (Latitude 8.9°N, Longitude 50°61 E). Omu-Aran is a town in the derived savannah ecological zone of North-Central Nigeria and is characterized by an annual rainfall between 600 and 1500 mm spreading between April and October with a peak usually in May to June and September to October. Whereas, the town experiences its dry season between November and March. The farm used for this experiment has been used for continuous cropping since 2010 and the predominant vegetation is composed of weeds such as *Rottboellia cochinchinensis,* (Lour) Claton) (Itch grass), *Eleusine indica* (L.) Gaertn) (Goosegrass), *Echinochloa colona* (L.) Link) (Sour millet), *Euphorbia heterophylla* (L) (Milkweed) and *Ageratum conyzoides* (L.) (Goat weed).

### Organic fertilizer application rates determination

A standard method of 10 kg N/ha was adopted in applying the fertilizer^49^. From this application rate, five other rates were calculated. The application rate then followed the following order for six different runs subsequently performed:

Total Nitrogen (N) in the papaya fruit peels organic fertilizer is 0.334 mg/g.

1 $$ {\text{Conversion}}\,{\text{of}}\,{\text{this}}\,{\text{value}}\,{\text{to}}\,{\text{percentage}}\,\left( {\frac{0.334 \times 100}{{1000}}} \right)\,{\text{which}}\,{\text{gave}}\,0.03\% $$

Using 10 kg N/ha as standard for 10 kg of soil in pot experiments, the quantity of papaya fruit peels organic fertilizer needed was:

2 $$ \frac{100}{{0.03}} \times 10 \times \frac{10}{{2,000,000}} \times 1000\,{\text{g}} $$

This gave 166.7 g of papaya fruit peels organic fertilizer to 10 kg of soil. Where one hectare of land contains 2,000,000 kg of soil as standard^50^, the papaya fruit peel organic fertilizer was converted to grams by multiplying by 1000. Therefore, total papaya fruit peel organic fertilizer needed per ha of crop field was calculated to be:

3 $$ \frac{166.7}{{10}} \times 2,000,000\,{\text{g}}/{\text{ha}} = 33,340,000\,{\text{g}}/{\text{ha}}\,{\text{or}}\,33.340\,{\text{kg}}/{\text{h}} $$

This gives approximately 33.340 kg/ha of papaya fruit peel organic fertilizer. Table 1 shows the quantity of papaya fruit peel organic fertilizer that was used for six different application rates (10, 20, 30, 40, 50, and 60) kg N/ha.

**Table 1:** Quantity of *Carica papaya* fruit peel organic fertilizer applied.

S/N	Experiments	Fertilizer quantity needed	Application rate (kg N/ha)
1	Negative Control	No fertilizer	0
2	NPK 15: 15: 15	66.7 g	26,680
3	10 kg N/ha	83.3 g	33,320
4	20 kg N/ha	166.6 g	66,640
5	30 kg N/ha	249.9 g	99,960
6	40 kg N/ha	333.2 g	132,880
7	50 kg N/ha	416.5 g	166,600
8	60 kg N/ha	499.8 g	199,920

### Soil preparation

Low nutrient (< 5% Nitrogen content) sand-loamy soil (Ultisols) as shown in Table 2 was used in the pot experiments to ascertain the nutrient status of the organic fertilizer. A bulk soil sample was collected during the cropping seasons to determine the physicochemical properties. This was done after the land was mechanically prepared with the aid of a tractor-drawn disc plough and harrow. Ploughing was carried out once while harrowing was twice and the resulting soil was well pulverized. Thereafter, a randomized complete block experimental design was adopted using experimental pots with 4 replicates for each treatment. The size for each plot was 2 m × 2 m) which equals 4m^2^ and this gave a net plot size of 28 m × 4 m) which equals 122 m^2^. Each experimental pot was filled with 10 kg of soil to which was added the measured organic fertilizers and thoroughly mixed before a two-week incubation for proper mixing and mineralization before planting of maize seeds. In the design, each organic fertilizer application was repeated every 15-day till fruit maturity and harvesting. The NPK 15–15–15 inorganic fertilizer (Positive control) was manually applied two weeks after sowing by side placement at 5 to 8 cm distance from the base of the plant while the other experiment was without fertilizer application and served as a negative control. Physicochemical and microbial analyses were performed on soils collected before planting and after crop harvesting. Weeding was manually done by hand-picking of weeds at 15 days after sowing (DaS) and was repeated every other 15 days.

**Table 2:** Soil physicochemical properties and microbial composition.

Chemical properties		Microbial composition			
Parameter	Soil (mg/L)	Bacteria		Fungi	
		Organism	TBPC (cfu/ml)	Organism	TFC (cfu/ml)
Nitrogen (N)	9.2	*Bacillus* sp.	4.1 × 10^5^	*Aspergillus niger*	3.0 × 10^3^
Phosphorus (P)	1.4			*Mucor* sp.	
Potassium (K)	2.6	*Clostridium* sp.			
Calcium (Ca)	43.3				
Magnesium (Mg)	21.6				
Copper (Cu)	1.25				
Zinc (Zn)	10				
Iron (Fe)	2.1				
Aluminium (Al)	0.05				
Nitrate (NO_3_)	1.09				
Ammonium (NH_4_)	0.21				
Phosphate (PO_4_)	44.4				
Manganese (Mn)	0.008				
Sulphate (SO_4_)	41.5				

*TBPC* total bacterial plate count, *TFC* total fungal count.

### Planting and data collection

An extra early maturing maize hybrid (Ife Maizehyb-5) from the International Institute of Tropical Agriculture (IITA) was used in this experiment. Seed viability testing was carried out by 24 h soaking in distilled water 30 °C. Viable seeds sat at the bottom of the beaker and these were used giving 90% germination percentage when used for the experiments^51^. In each pot, maize seeds were sown with a planting distance of 75 × 25 cm, and the Phyto-parameters data was collected at 15-day intervals after the emergence of seeds as shown in Table S1 (Supplementary materials). These measurements of plant parameters were terminated at 50 (DaS); before tasselling when the optimal nutrient uptake had taken place before the commencement of the generative phase. Nutrient analysis taken after this period i.e. 60–75 DaS were intended to measure the leftover nutrients after the photosyntate have been mobilized to tassel, ear, corncob, and grain. Measurement of the last 4 parameters (shoot and root biomass, root length, and total ear size) were carried out after crop harvesting. The nutrients content accumulated in the plant’s leaf, stem, and root was analyzed by using the method described in Analytical procedures section.

#### Statistical analyses

Analyses were carried out using the Analysis of variance (ANOVA) while a comparison of mean was done with Tukey's test. All statistical analyses were performed using the Statistical Analysis Software (SAS/STAT), Version 8, 6th eds.

## Results

### Effect of pretreatment and AD on *C. papaya* fruit peel

Table 3 shows the results of physicochemical characteristics of the papaya fruit peel and inoculum used in the production of the organic fertilizer in this study. As seen from the table, the pH of the reactor was slightly alkaline throughout the digestion period after experiencing an initial fall during the first 5 days. In the same way, the temperature was kept at the mesophilic range all through the digestion. It is evident from Table 3 that the thermo-alkaline pretreatment applied to the biomass caused the solubilization of important structural components especially the lignin-cellulose-hemicellulose matrix. This effect was further compounded during AD as the condition of digestion also imparted on further degradation of the substrate thereby yielding nutrients that was initially locked up in the biomass. As a result, the increment was recorded for most chemical parameters except for five of them i.e., total solids, volatile solids, carbon, calcium, and COD. Therefore, the anaerobic digestates obtained were nutrient-rich.

**Table 3:** Physical and chemical characteristics of *Carica papaya* fruit peel and cattle rumen content^38^.

Parameters	Rumen content	Raw *C. papaya* peel only	Pretreated *C. papaya* fruit peel + rumen content	
			Before digestion	Raw digestate before dewatering
pH	7.91 ± 0.02	6.23 ± 1.00	7.70 ± 0.02	7.60 ± 0.03
Total Solids (g/kg TS)	90.52 ± 0.11	94.81 ± 1.21	110.97 ± 0.11	93.94 ± 0.02
Volatile Solids (g/kg TS)	80.44 ± 1.12	83.23 ± 0.22	96.22 ± 3.02	50.01 ± 2.02
Ash Content (%)	5.56 ± 1.02	2.54 ± 1.00	2.78 ± 0.00	5.49 ± 0.03
Moisture Content (%)	90.48 ± 3.02	97.26 ± 0.01	94.03 ± 4.01	96.06 ± 1.02
Chemical Oxygen Demand (mg/kg TS)	168.21 ± 1.12	165.11 ± 2.20	256.5 ± 4.04	83 ± 2.01
Total Carbon (g/kg TS)	265.21 ± 0.10	202.90 ± 4.03	214.90 ± 5.03	200.10 ± 3.03
Total Nitrogen (g/kg TS)	48.00 ± 2.02	37.51 ± 2.02	40.00 ± 1.01	41.60 ± 0.11
Total Phosphorus (g/kg TS)	6.30 ± 0.02	5.32 ± 1.02	6.12 ± 0.01	7.60 ± 1.11
Potassium (g/kg TS)	7.20 ± 0.11	7.32 ± 2.00	8.00 ± 0.11	10.94 ± 0.03
Phosphate (g/kg TS)	3.00 ± 0.02	1.03 ± 0.11	3.00 ± 0.10	4.51 ± 0.02
Sulfate (g/kg TS)	134 ± 2.00	112.20 ± 3.01	136.00 ± 2.03	159.49 ± 0.03
Calcium (g/kg TS)	80.00 ± 0.10	220.81 ± 4.41	226.00 ± 4.09	89.06 ± 2.00
Magnesium (g/kg TS)	96.00 ± 0.10	89.32 ± 1.02	100.00 ± 0.03	200.10 ± 5.05
Manganese (g/kg TS)	1.18 ± 0.22	0.021 ± 1.00	0.028 ± 0.00	0.060 ± 0.01
Iron (g/kg TS)	1.18 ± 0.11	1.06 ± 0.11	1.16 ± 0.21	4.60 ± 1.00
Zinc (g/kg TS)	38.00 ± 0.02	32.32 ± 0.01	36.00 ± 0.03	40.94 ± 1.22
Aluminium (g/kg TS)	0.80 ± 0.11	0.52 ± 1.02	0.76 ± 0.02	0.91 ± 0.03
Copper (g/kg TS)	4.80 ± 0.10	3.87 ± 0.03	4.70 ± 0.03	5.49 ± 0.03

### Physicochemical compositions of inorganic fertilizer and organic fertilizer

**Figure 2:**
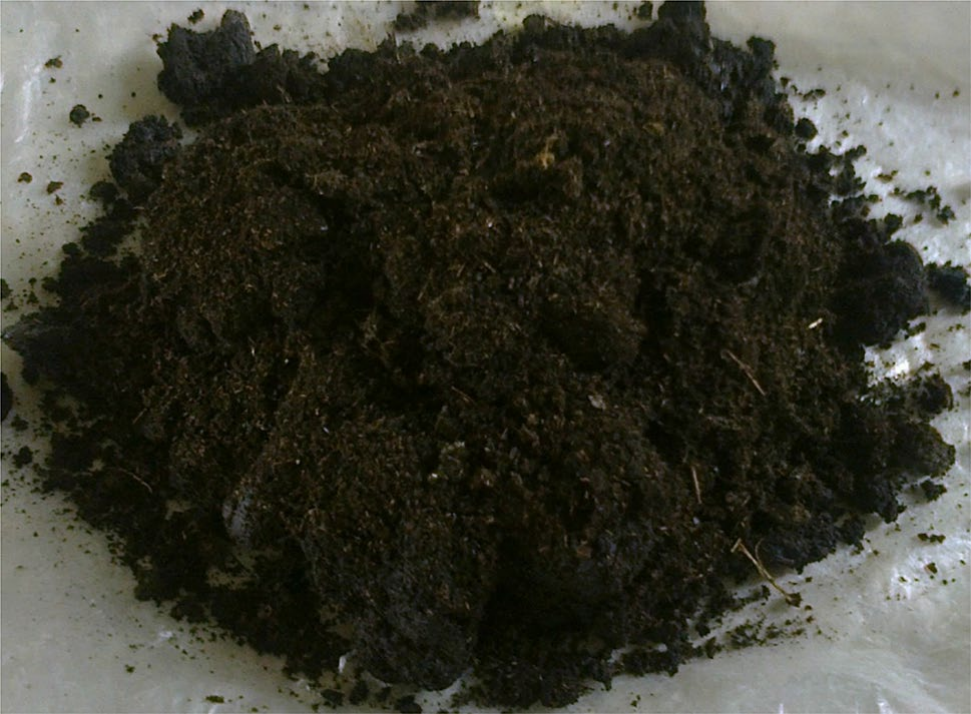
The organic fertilizer produced and used in this study.

The papaya fruit-peel-based organic fertilizer produced by the AD method is shown in Fig. 2. After dewatering, the organic fertilizer and the inorganic fertilizer were evaluated for major and minor nutrients/elements including pH as shown in Table 4. Calcium has the highest concentration in the organic fertilizer with an average value of 14.00% while manganese had the lowest value of 0.003%. The composition of the inorganic fertilizer used as control is shown in Table S2 (Supplementary materials).

**Table 4:** Mineral composition of *Carica papaya* (Pawpaw) fruit peels organic fertilizer.

S/N	Parameter	Composition (%)
1	pH	7.30 ± 0.01
2	Copper	0.65 ± 0.02
3	Calcium	14.00 ± 0.02
4	Iron	0.14 ± 0.01
5	Magnesium	4.80 ± 0.02
6	Manganese	0.003 ± 0.01
7	Phosphate	0.30 ± 0.01
8	Sulfate	1.94 ± 0.05
9	**Potassium**	**1.22** ± 0.02
10	**Nitrogen**	**9.18** ± 0.03
11	**Phosphorus**	**0.62** ± 0.01
12	Zinc	3.46 ± 0.01
13	Aluminium	0.08 ± 0.01

Values shown in table are means of triplicate analyses.

### Microbial evaluation of anaerobic digestate and organic fertilizer

The microbial composition of the digestate and the dewatered organic fertilizer are shown in Table 5. The microbial diversity and populations in the digestate far exceed those of the organic fertilizer. The total bacterial count of the digestate and organic fertilizer was 2.4 × 10^12^ and 9.0 × 10^6^ CFU/ml respectively while the total fungal counts were 4.0 × 10^4^ and 4.0 × 10^2^ CFU/ml*.*

**Table 5:** Microbial composition of *Carica papaya* fruit peel organic fertilizer.

Before dewatering		After dewatering		Before dewatering		After dewatering	
Bacteria	TBPC (cfu/ml)	Bacteria	TBPC (cfu/ml)	Fungi	TFC (cfu/ml)	Fungi	TFC (cfu/ml)
*Bacillus* sp. *Enterococcus* sp. *Pseudomonas aeruginosa* *Proteus* sp. *Fusobacterium* sp. *Bacteroides fragilis Clostridium* sp. *Gemella* sp. *Methanococcus* sp. *Methanosaeta* sp. *Methanobacteriales* sp.	2.4 × 10^12^	*Bacillus* sp. *Enterococcus* sp. *Pseudomonas aeruginosa* *Proteus* sp. *Fusobacterium* sp. *Bacteroides* sp. *Clostridium* sp. *Gemella* sp.	9.0 × 10^6^	*Aspergillus niger* *Mucor* sp. *Rhizopus* sp. *Penicillum* sp.	4.0 × 10^4^	*Aspergillus niger* *Mucor* sp. *Rhizopus* sp. *Penicillum* sp.	4.0 × 10^2^

Values shown in table are means of triplicate analyses; *TBPC* total bacterial plate count, *TFC* total fungal count.

### Field assessment result

Table 6 shows the results of the field assessment from the control experiments. In the no fertilizer application, the number of leaves, leaf area, plant height, and stem girth all increased with progress between 15 and 75 DaS and the highest values of 17 leaves, 99.7 cm^2^, 165 cm, and 2.8 cm were for the four parameters at the end of the experiments. After harvesting, the average total shoot biomass was 221 g, while that of the root was 59.1 g. The total root length was 23 cm while the average total size of the harvested ear was 316.3 g. From the NPK 15–15–15 treated plot, the same trend was recorded for the number of leaves, leaf area, plant height, and stem girth which all increased with progress in the experiment with the highest values recorded as 17 leaves, 104.1 cm^2^, 142 cm, and 2.9 cm for the four parameters respectively. After harvesting, the average total shoot biomass was 255 g, while that of the root was 63 g. The total root length was 26 cm while the average total size of the harvested ear was 325.7 g.

**Table 6:** Results of Phyto-Assessment in control, NPK 15–15–15 and *Carica papaya* fruit peel organic fertilized experiments using Maize (*Zea mays*) as test plant.

DAE	Leaf number	Leaf area (cm^2^)	Plant height (cm)	Stem girth (cm)	Biomass above soil level (g)	Root biomass (g)	Root length (cm)	Total ear size (g)
**No Fertilizer Application**								
15	5 ± 0.01^a^	22.9 ± 0.02^a^	22 ± 1.01^a^	0.7 ± 0.01^a^	–	–	–	–
30	7 ± 0.01^a^	45.1 ± 0.01^a^	42 ± 0.02^a^	0.9 ± 0.01^a^	–	–	–	–
45	10 ± 0.01^a^	62.4 ± 0.01^a^	85 ± 1.01^a^	1.4 ± 0.01^a^	–	–	–	–
60	15 ± 0.01^a^	87.4 ± 0.01^a^	148 ± 1.01^a^	2.1 ± 0.01^a^	–	–	–	–
75	17 ± 0.01^a^	99.7 ± 0.01^a^	165 ± 0.01^a^	2.8 ± 0.01^a^	221 ± 0.01^a^	59.1 ± 0.01^a^	23 ± 2.01^a^	316.3 ± 4.01^a^
**NPK 15–15–15 Fertilizer Application (30 kg N/ha)**								
15	5 ± 0.01^a^	23.5 ± 0.01^a^	18 ± 0.01^b^	0.7 ± 0.01^a^	–	–	–	–
30	6 ± 0.01^b^	46.2 ± 0.01^b^	35 ± 0.03^b^	1.3 ± 0.01^b^	–	–	–	–
45	10 ± 0.01^a^	74.5 ± 0.01^b^	74 ± 0.02^b^	1.9 ± 0.01^b^	–	–	–	–
60	16 ± 0.01^b^	94.9 ± 0.01^b^	121 ± 0.03^b^	2.4 ± 0.01^b^	–	–	–	–
75	17 ± 0.01^a^	104.1 ± 0.01^b^	142 ± 0.02^b^	2.9 ± 0.01^a^	255 ± 0.05^b^	63 ± 0.02^b^	25 ± 1.01^a^	325.7 ± 5.01^b^
**10 kg N/ha Application**								
15	4 ± 0.01^b^	20.1 ± 0.01^b^	15 ± 0.01^c^	0.6 ± 0.01^a^	–	–	–	
30	8 ± 0.01^c^	39.2 ± 0.01^c^	31 ± 2.01^c^	1.2 ± 0.01^b^	–	–	–	
45	11 ± 0.01^b^	70.5 ± 0.02^c^	76 ± 2.01^b^	1.6 ± 0.01^c^	–	–	–	
60	18 ± 0.01^c^	90.5 ± 0.01^c^	91 ± 2.01^c^	1.9 ± 0.01^c^	–	–	–	
75	18 ± 0.01^b^	107 ± 0.02^b^	130 ± 2.01^c^	2.7 ± 0.01^a^	225 ± 4.01^a^	60 ± 3.01^b^	21 ± 1.01^a^	253 ± 5.05^c^
**20 kg N/ha Application**								
15	6 ± 0.01^c^	30.1 ± 1.01^c^	18 ± 2.01^b^	1.1 ± 0.01^b^	–	–	–	
15	9 ± 0.01^d^	50.1 ± 2.01^d^	36 ± 2.01^b^	1.3 ± 0.01^b^	–	–	–	
45	12 ± 0.01^c^	86.1 ± 2.01^d^	86 ± 2.01^a^	1.8 ± 0.01^b^	–	–	–	
60	17 ± 0.01^d^	101 ± 2.01^d^	103 ± 2.01^d^	2.1 ± 0.01^a^	–	–	–	
75	18 ± 0.01^b^	127 ± 3.01^c^	147 ± 4.01^d^	2.9 ± 1.01^a^	219 ± 5.02^a^	65.5 ± 2.01^b^	22 ± 0.05^a^	259 ± 6.03^c^
**30 kg N/ha Application**								
15	7 ± 0.01^d^	30.8 ± 1.01^c^	16 ± 0.03^c^	1.4 ± 0.01^c^	–	–	–	
30	9 ± 0.01^d^	50.8 ± 2.01^d^	63 ± 1.04^d^	1.9 ± 0.01^c^	–	–	–	
45	12 ± 0.01^c^	90.8 ± 2.01^e^	83 ± 2.04^a^	2.3 ± 0.01^d^	–	–	–	
60	*19* ± *0.01*^e^	120.0 ± 3.01^e^	134 ± 3.04^e^	2.8 ± 0.01^d^	–	–	–	
75	*19* ± *0.01*^c^	*150.3* ± *0.01*^d^	155 ± 4.05^e^	3.1 ± 0.01^b^	*424* ± *5.05*^c^	*146* ± *4.05*^c^	*25.5* ± *2.01*^a^	*321* ± *6.03*^b^
**40 kg N/ha Application**								
15	7 ± 0.01^d^	30.7 ± 1.01^c^	17 ± 2.01^b^	1.6 ± 0.01^d^	–	–	–	
30	9 ± 1.01^d^	50.2 ± 1.01^d^	60 ± 2.01^e^	2.4 ± 0.01^d^	–	–	–	
45	13 ± 0.01^d^	80.2 ± 2.01f.	85 ± 2.01^a^	2.5 ± 0.01^e^	–	–	–	
60	*19* ± *0.01*^e^	104.5 ± 1.01^d^	132 ± 3.04^e^	2.8 ± 0.01^d^	–	–	–	
75	*19* ± *1.01*^c^	147.2 ± 2.01^d^	159 ± 3.01f.	3.4 ± 0.01^c^	393.5 ± 3.01^d^	139.5 ± 4.01^c^	23 ± 2.00^a^	312 ± 2.05^d^
**50 kg N/ha Application**								
15	7 ± 0.01^d^	30.8 ± 0.01^c^	16 ± 2.01^c^	1.6 ± 0.01^d^	–	–	–	
30	9 ± 0.02 ^d^	51.0 ± 1.02^d^	62 ± 1.04^d^	2.6 ± 0.01^e^	–	–	–	
45	13 ± 0.02^d^	83.2 ± 2.02f.	87 ± 1.04^c^	2.9 ± 0.01f.	–	–	–	
60	*19* ± *0.01*^e^	101.5 ± 2.01^d^	152 ± 2.04f.	3.2 ± 0.01^e^	–	–	–	
75	*19* ± *0.01*^c^	140.6 ± 4.01^e^	157 ± 2.01f.	*3.5* ± *0.01*^c^	418 ± 3.01^c^	144 ± 2.01^c^	25.1 ± 0.05^a^	316 ± 5.03^d^
**60 kg N/ha Application**								
15	7 ± 0.01^d^	40.0 ± 0.01^d^	17 ± 0.01^b^	1.5 ± 0.01^d^	–	–	–	
30	9 ± 0.03^d^	50.4 ± 1.02^d^	66 ± 2.01f.	2.8 ± 0.01f.	–	–	–	
45	13 ± 0.03^d^	90.1 ± 2.02^e^	96 ± 2.01^d^	2.9 ± 0.01f.	–	–	–	
60	*19* ± *0.01*^e^	114.5 ± 2.01f.	158 ± 2.03^ g^	3.3 ± 0.01^e^	–	–	–	
75	*19* ± *0.01*^c^	144.7 ± 3.01^e^	*173* ± *3.01*^ g^	*3.5* ± *0.01*^c^	414.5 ± 3.01^c^	117.5 ± 2.0^d^	24.5 ± 2.05^a^	309 ± 4.05^e^

Values shown in table are means of triplicate analyses; DAE = Day after Emergence; italics values are the highest obtained for each phyto-parameter; superscripts with same letters are statistically the same for a particular parameter across the different fertilizer treatments (Organic fertilizer and control experiments) by the Tukey’s test at 5%.

**Figure 3:**
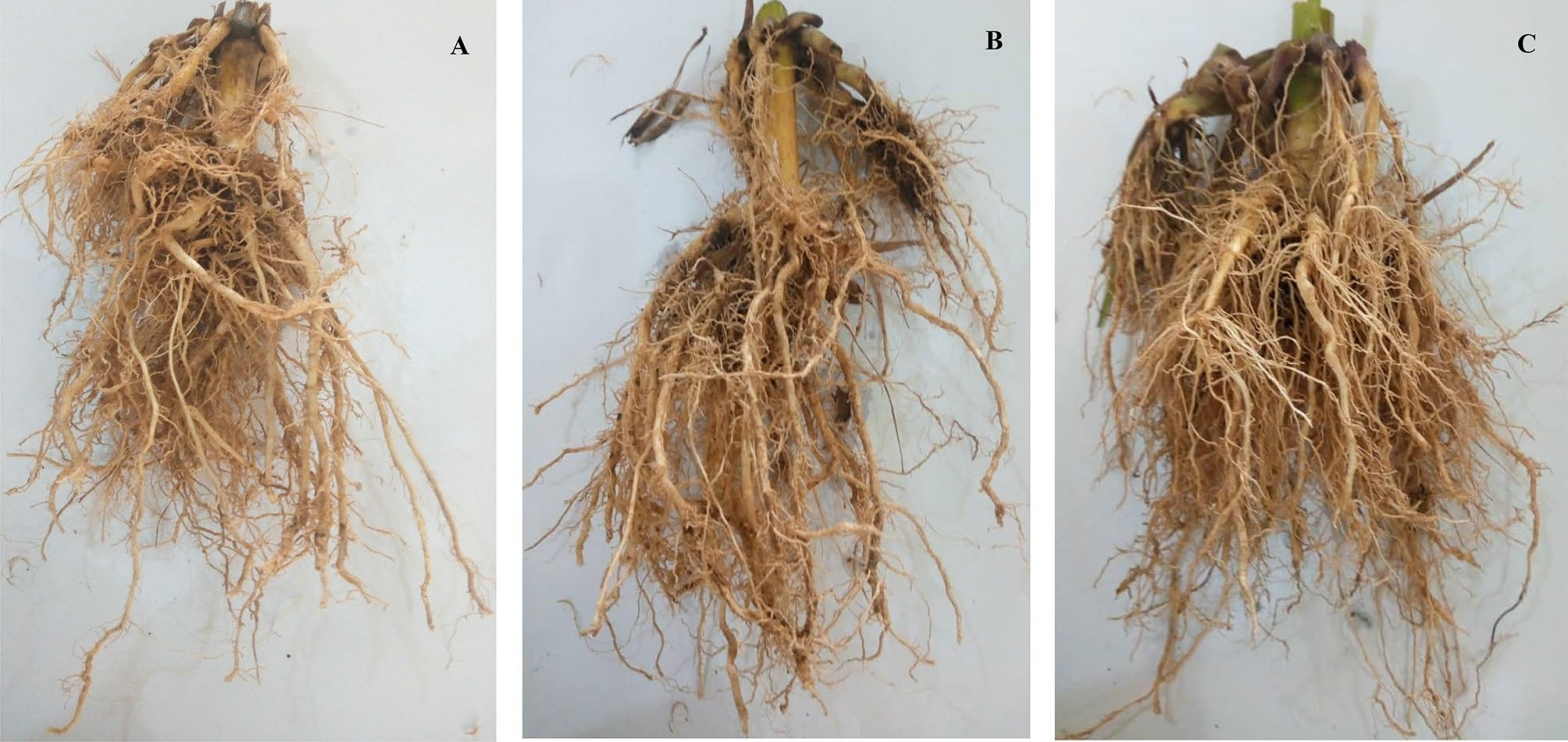
Complete root system from (**A**) the control (No fertilizer application) experiment (**B**) the NPK fertilized experiment (**C**) organic fertilized experiments (30 kg N/ha).

Table 6 further shows the result of the field assessments with papaya fruit peel organic fertilizer. From these experiments, there was an increase in values of all parameters corresponding to the progress of the experiment. The highest number of leaves (19) was recorded in the maize treated with 30, 40, 50, and 60 kg N/ha organic fertilizer application and recorded at 60 and 75 days after the emergence of seed. The highest leaf area (150.3 cm^2^) was recorded in the 30 kg N/ha experiment, while those of plant height (173 cm) and stem girth (3.5 cm) were both recorded in the 60 kg N/ha experiment at the end of the experimental period. The highest weight of shoot and root biomass were 424 146 g respectively both recorded in the 30 kg N/ha experiment after crop harvesting. The highest root length (25 cm) was recorded in the 30 kg N/ha experiment while the average size of the harvested ear was 321 g also from the experiment with 30 kg N/ha organic fertilizer application. In general, the organic fertilizer showed better performance in all parameters such as the number of leaves, leaf area, plant height, stem girth, total shoot, and root biomass, and length of the root. However, the chemical fertilizer outperformed all the organic fertilizer applied rates in the average highest size of the corn ear by 1.4% i.e. 325.7 and 321 g for chemical fertilizer and organic fertilizer (30 kg N/ha) respectively. Comparison between the values obtained from all the field experiments involving papaya fruit peel organic fertilizer and the controls reveals statistical difference at 5% confidence interval for all field parameters except the root length with values that are all statistically the same. Figure 3 shows the root images from the different treatments i.e., Fig. 3A is the root system of a representative from the control (No fertilizer application) experiment, Fig. 3B represents the root system of an NPK fertilized maize plant while Fig. 3C represents the root system of a 30 kg N/ha organically fertilized experiment. On the other hand, Figs. S1 and S2 (Supplementary materials) show the complete shoot system of representative plants from the controls i.e. No fertilizer application and NPK 15–15–15 fertilized experiments and the 30 kg N/ha organically fertilized experiment.

### Nutrient bioavailability and uptake

Table 7 shows the results of the accumulation of three major nutrient elements (N, P, K) in the leaves, stems, and roots of the maize plants from the control experiments. In the negative control experiments, nitrogen, phosphorus, and potassium had their highest concentrations of 20.5, 2.92, and 3.5 mg/L respectively in the plant roots while in the positive control experiment, all the 3 major elements (NPK) had their highest concentrations of 16.8, 1.9 and 3.3 mg/L respectively in the plant stem.

**Table 7:** Nutrient bioavailability and accessibility to plant organs in control, NPK 15–15–15 and *Carica papaya* fruit peel organic fertilized experiments using Maize (*Zea mays*) as test plant (Measured at 75 DAE).

Nutrient (mg/L)	Leaves	Stem	Roots
**No Fertilizer application**			
Nitrogen (N)	17 ± 1.02^a^	18.1 ± 2.02^a^	20.5 ± 2.01^a^
Phosphorus (P)	2.11 ± 0.01^a^	2.22 ± 0.03^a^	2.92 ± 0.02^a^
Potassium (K)	3.0 ± 0.01^a^	3.2 ± 0.02^b^	3.5 ± 0.01^a^
**NPK 15–15–15 fertilizer application**			
Nitrogen (N)	15.5 ± 0.11^a^	16.8 ± 0.11^b^	15.8 ± 1.01^b^
Phosphorus (P)	1.58 ± 0.10^a^	1.9 ± 0.02^a^	1.82 ± 0.01^b^
Potassium (K)	2.9 ± 0.01^a^	3.3 ± 0.02^a^	3.2 ± 0.01^a^
**10 kg N/ha application**			
Nitrogen (N)	27.0 ± 5.02^b^	26.1 ± 4.02^c^	26.2 ± 3.05^c^
Phosphorus (P)	6.76 ± 1.01^b^	6.51 ± 0.01^b^	6.00 ± 1.01^c^
Potassium (K)	7.1 ± 2.01^b^	7.1 ± 0.01^b^	7.1 ± 2.01^b^
**20 kg N/ha application**			
Nitrogen (N)	28.0 ± 3.03^b^	27.1 ± 2.02^c^	27.4 ± 3.01^c^
Phosphorus (P)	4.77 ± 0.12^c^	4.52 ± 0.11^c^	5.05 ± 1.00^d^
Potassium (K)	7.2 ± 1.01^b^	7.3 ± 1.01^b^	7.5 ± 0.01^c^
**30 kg N/ha application**			
Nitrogen (N)	25.9 ± 3.01^c^	29.0 ± 5.01^d^	27.5 ± 3.04^c^
Phosphorus (P)	5.62 ± 0.02^d^	5.36 ± 0.01^d^	4.89 ± 1.01^d^
Potassium (K)	7.0 ± 1.01^b^	7.5 ± 1.01^c^	7.4 ± 0.02^c^
**40 kg N/ha application**			
Nitrogen (N)	29.0 ± 4.01^b^	27.1 ± 4.01^c^	27.4 ± 3.02^c^
Phosphorus (P)	4.79 ± 1.01^c^	4.53 ± 0.03^c^	5.15 ± 1.01^e^
Potassium (K)	7.5 ± 1.01^c^	7.3 ± 2.00^b^	7.5 ± 1.01^c^
**50 kg N/ha application**			
Nitrogen (N)	29.7 ± 2.01^d^	27.6 ± 4.01^c^	28.0 ± 1.01^c^
Phosphorus (P)	4.77 ± 1.01^c^	*7.05* ± *1.01*^c^	4.51 ± 1.01^c^
Potassium (K)	7.3 ± 1.02^c^	**8.4** ± **2.01**^b^	7.1 ± 2.01^b^
**60 kg N/ha application**			
Nitrogen (N)	26.8 ± 3.03^c^	29.0 ± 4.01^d^	27.1 ± 3.03^c^
Phosphorus (P)	4.01 ± 1.01^e^	5.19 ± 2.01^e^	5.01 ± 1.01^d^
Potassium (K)	6.9 ± 2.01^b^	7.6 ± 0.03^c^	7.4 ± 2.01^c^

Values shown in table are means of triplicate analyses; superscripts with same letters across each plant organ for the different fertilizer treatment treatments (Organic fertilizer and control experiments) are statistically the same by Tukey’s test at 5%; Value in underline indicates highest concentration of Nitrogen in the leaf, while those in italics and bold indicates highest levels of phosphorus and potassium respectively in the root.

The results of the accumulation of nutrient elements in the leaves, stems, and roots of the maize plants from the organic fertilized experiments are also presented in Table 7. Nitrogen recorded its highest concentrations of 29.7 mg/L in the leaf and this value was reported from the experiment with 50 kg N/ha. For phosphorus and potassium, the highest concentrations of 7.05 and 8.4 mg/L were recorded in the plant’ stem of the experiment with 50 kg N/ha. Overall, the stem displayed the highest ability to store nutrient elements, and the 50 kg N/ha experiment showed the highest level of nutrient bioavailability. As shown in Table 7, statistical comparison between the values obtained from all the nutrient bioavailability experiments involving papaya fruit-peel-based organic fertilizer and the controls reveals statistical difference at a 5% confidence interval for all parameters across all treatments.

### Soil fertility improvement assessment

As shown in Table 2, the chemical and microbial compositions of the experimental soil show that it is of low nutrient status and also low in microbial composition when compared to fertile soils as seen from the table. The total bacterial count was 4.1 × 10^5^ CFU/ml while the fungal count was 3.0 × 10^3^ CFU/ml. As shown in Table 8, the application of papaya fruit-peel-based organic fertilizer improved soil fertility in the long run. The lowest recorded fertility enhancement was found in the negative control and highest in the experiment with 60 kg N/ha. After plant harvesting, nitrogen, phosphorus, and potassium increased in experimental soils by 28, 40, and 22% in the experiment with 60 kg N/ha over the chemical fertilizer applied experiment, and the same trend was recorded for all other parameters as shown in the table. All the values recorded across the different treatments (Papaya fruit peel organic fertilizer and the controls) reveal statistical differences at a 5% confidence interval.

**Table 8:** Soil fertility improvement by *Carica papaya* fruit peel organic fertilizer.

Parameters (mg/L)	Control	NPK	10 kg N/ha	20 kg N/ha	30 kg N/ha	40 kg N/ha	50 kg N/ha	60 kg N/ha
Nitrogen (N)	10.0 ± 0.02^a^	27.6 ± 2.02^b^	30.2 ± 0.01^c^	36.2 ± 0.01^d^	36.7 ± 0.12^d^	31.9 ± 0.02^b^	37.6 ± 1.03^d^	*38.2* ± *0.02*^d^
Phosphorus (P)	1.1 ± 0.01^a^	3.8 ± 0.02^b^	5.5 ± 0.01^c^	5.6 ± 0.02^c^	5.8 ± 0.02^c^	6.9 ± 0.02^d^	6.2 ± 0.02^c^	*6.3* ± *0.02*^c^
Potassium (K)	1.6 ± 0.01^a^	5.7 ± 0.01^b^	6.5 ± 0.02^b^	6.6 ± 0.02^b^	6.7 ± 0.02^b^	6.9 ± 0.02^b^	7.1 ± 0.02^b^	*7.3* ± *0.03*^b^
Calcium (Ca)	36.2 ± 2.02^a^	74.4 ± 2.02^b^	86.5 ± 2.01^c^	87.5 ± 0.02^c^	89.5 ± 0.05^c^	98.3 ± 0.02^d^	93.6 ± 0.03^d^	*106.2* ± *0.02*^e^
Magnesium (Mg)	21.2 ± 1.01^a^	46.3 ± 1.02^b^	55.5 ± 0.03^c^	56.5 ± 0.03^c^	56.6 ± 0.01^c^	58.4 ± 0.02^c^	61.5 ± 0.02^c^	*63.3* ± *0.03*^c^
Copper (Cu)	1.01 ± 0.02^a^	2.0 ± 0.02^b^	1.95 ± 0.02^b^	2.8 ± 0.02^c^	3.1 ± 0.02^c^	3.1 ± 0.03^c^	4.5 ± 0.02^d^	*4.7* ± *0.02*^d^
Zinc (Zn)	6.9 ± 1.02^a^	16 ± 1.02^b^	14.5 ± 0.02^b^	16 ± 0.01^b^	21.5 ± 0.01^c^	21.7 ± 0.01^c^	23 ± 1.02^c^	*23.5* ± *1.02*^c^
Iron (Fe)	1.6 ± 0.01^a^	3.5 ± 0.02^b^	3.5 ± 0.02^b^	4.1 ± 0.02^b^	4.5 ± 0.02^c^	4.7 ± 0.02^c^	4.9 ± 0.02^c^	*6.2* ± *0.02*^d^
Aluminium (Al)	0.11 ± 0.02^a^	0.43 ± 0.02^b^	0.41 ± 0.02^b^	0.52 ± 0.02^c^	0.54 ± 0.02^c^	0.56 ± 0.02^c^	0.65 ± 0.02^d^	*0.68* ± *0.02*^d^
Nitrate (NO_3_)	0.4 ± 0.02^a^	1.6 ± 0.02^b^	1.4 ± 0.01^b^	1.45 ± 0.01^b^	1.55 ± 0.00^c^	1.52 ± 0.01^c^	1.7 ± 0.02^d^	*2.0* ± *0.02*^d^
Ammonium (NH_4_)	0.11 ± 0.01^a^	0.36 ± 0.02^b^	0.35 ± 0.02^b^	0.35 ± 0.02^b^	0.36 ± 0.01^b^	0.37 ± 0.12^b^	0.35 ± 0.05^b^	*0.39* ± *0.02*^c^
Phosphate (PO_4_)	43.2 ± 1.02^a^	75.6 ± 4.02^b^	75.4 ± 1.02^b^	76.1 ± 1.02^b^	77.5 ± 1.02^b^	78.3 ± 2.01^b^	83.2 ± 2.01^b^	*84.5* ± *1.00*^b^
Manganese (Mn)	0.006 ± 0.01^a^	0.013 ± 0.02^a^	0.012 ± 0.01^b^	0.012 ± 0.01^b^	0.013 ± 0.01^b^	0.014 ± 0.01^b^	0.014 ± 0.01^b^	*0.018* ± *0.01*^c^
Sulfate (SO_4_)	34.4 ± 1.02^a^	62.5 ± 2.02^b^	61.2 ± 2.03^b^	61.1 ± 2.02^b^	62.5 ± 2.01^b^	62.5 ± 0.02^b^	64.2 ± 1.02^b^	*67.5* ± *2.03*^c^

Values shown in table are means of triplicate analyses; superscripts with same letters are statistically the same by the Tukey’s test at 5%; Values in italics indicates highest concentrations of each parameter measured.

## Discussion

### Effects of pretreatment on Papaya fruit peel

The papaya fruit peel used in this study was solubilized with evidence of a breakdown in the lignin-cellulose-hemicellulose complex and other important structural materials after pretreatment with heat and chemical application. Heating at 80 °C ensured the stabilization of the resulting substrate as against the use of higher temperatures which has been reported to either cause excess solubilization or the production of complex proteins that in turn hinders AD and ultimately affect the quality of produced digestate^28–32,52,53^. These pretreatments have also been applied to several lignocelluloses and the result was a structural component breakdown and high product yield^54,55^.

### Physical parameters of biomass

Different raw materials with suitable physical and rheological properties e.g. lignocellulosic biomass are cheap sources of digestate organic fertilizers, making them preferable to inorganic fertilizers due to production and supply costs^56,57^. This factor has over the last decades promoted the use of anaerobic digestates as organic fertilizers with its attendant reduction in chemical usage and its build-up in the food chain^58^. During the AD in this study, pH values are within the alkaline range which is very similar to earlier reports that alkaline pH range is best for microbial activities during digestion^25,28–30^ while lower pH ranges can negatively affect microbial functions and could disrupt the entire process^43,59^. In most studies, microbial abundance and activities have been closely linked to the alkaline pH range in the digester^60–65^. Thus, maintaining suitable pH during digestion is a vital condition in efficient bioconversion of substrate, digestion stability, and product yield enhancement^66,67^. Due to the enormous biochemical reactions during AD, the temperature is also very important because most bacteria and methanogens implicated in the process usually thrive at either mesophilic or thermophilic ranges^68,69^. The mesophilic temperature in this study contributed to digestion stability besides the provision of support for bacteria proliferation and activities^70,71^. A retention time (RT) of 30 ± 2 was employed in this study to provide proper ambiance for anaerobic microbes causing efficient digestion of the substrate. A similar result was earlier reported by Mao et al.^71^.

### Chemical parameters of biomass

Results show that the papaya fruit peel is composed of nutrients required by microbes for their growth in a fermentation medium. Besides, the rumen content used as inoculum is equally rich in nutrients and microbes^72,73^. The high nutrients and elemental content of papaya fruit peel are due to its nutrient storage ability especially in its epicarp coupled with variations in the season which most times determines the availability of nutrients to the plant. These qualities make papaya fruit peel better than other lignocellulosic biomass such as the fruit rind of fluted pumpkin (*Telfairia occidentalis*), Pineapple (*Ananas comosus*), Peanut (*Arachis hypog*aea) pod, Cocoa (*Theobroma cacao*) pod husk, Siam weed (*Chromolaena odorata*) and wild Mexican Sunflower (*Tithonia diversifolia*) shoots^28–31,33–36^ while if slightly differ from kitchen wastes and other animal-based wastes such as cow and piggery dungs. The use of nutrient-rich substrates such as papaya fruit peel in the AD process had been advocated^74^. The Nitrogen contents of the papaya fruit peel are suitable for an ideal AD substrate^74^.

### Organic fertilizer quality

The papaya peel-based anaerobic digestate obtained was richer in nutrients than the raw biomass. This trend was equally influenced by the application of pretreatments before AD. However, total solids, volatile solids, carbon, and calcium concentrations were reduced in the digestate as against their values in the raw pawpaw fruit peel and this can be attributed to the vital roles they play during microbial metabolism and for the synthesis of the microbial cell wall. The application of pretreatment coupled with high microbial population and activities made the pretreated biomass easily biodegraded with evidence of organic matter breakdown which led to COD reduction in the produced digestate. Application of pretreatments to lignocellulosic biomass before AD has been recommended to enhance ease of digestion and improvement in product quality^75–79^.

The digestate used in this study can therefore be adjudged to be nutrient-rich and is potent enough to improve the nutrient and microbial status of soil^80^. Besides soil improvement, this digestate when applied as organic fertilizer will have a great impact on the growth and health of plants especially in regions facing erosion of topsoil and depletion of nutrients. This corroborates the submission of previous studies on the use of AD digestates to supplement or sustainably replace chemical fertilizers due to the many environmental challenges posed by the latter in different cropping systems the world over^58,81–84^. Moreover, the microbial composition of the digestate produced in this study makes it potent to increase the population and diversity of beneficial microorganisms and suitable inoculants in the soil.

### Digestate microbial composition (before dewatering) and functions

During the evaluation of the digestate, different bacteria, fungi and methanogens were implicated most of which have earlier been reported to play important function during each stage of AD^68^ whose source is rumen content used as inoculum in this study. The dominant microbial group in digestate is the *Clostridia* which are common dwellers of the bovine rumen and are prominent amino-acid utilizers to produce volatile fatty acids (VFAs) with ammonia given off^85^. The population of facultative anaerobes was rather high in the digestate and this could be attributed to the alkaline nature of the digester which encouraged their proliferation and activities^33,34,71,86,87^. Besides the *Clostridia,* other prominent anaerobes *Fusobacterium mortiferum*, *Bacteroides fragilis,* and *Gemella morbillorum* all of which are regular inhabitants of anaerobic milieu such as AD. Similarly, the methanogens reported in the digestate are well known in the AD process^87^. Earlier, Dahunsi et al.^29^ had reported a rich microbial population is a major condition needed for improved degradation of substrates leading to the production of nutrient and microbe-rich digestates. Besides anaerobes, anaerobic digestates usually contain suitable aerobic microbes such as *Pseudomonas, Klebsiella, Aspergillus,* and *Bacillus* among others which are capable of quickening microbial processes in applied soils thereby increasing nutrients bioavailability to crop plants^88^.

### Microbial composition of organic fertilizer (dewatered digestate)

The higher composition of microbes in the digestate than the dewatered organic fertilizer is a result of pronounced water reduction in the organic fertilizer because the growth and activities of microbes are water-dependent. However, the organic fertilizer is very rich in microorganisms which are often employed as microbial inoculants for soil nutrients^88^. *Clostridium* and *Klebsiella species* are notable nitrogen-fixing bacteria in anaerobic conditions while *Bacillus species* are efficient at solubilizing phosphate^72^ and have also been implicated in nitrogen-fixing. These microbial processes often lead to nutrient availability in the organic fertilizer than the undigested or partially digested components and the overall effect of this is more efficient crops fertilization^89–91^. Besides, organic fertilizers provide ecological advantages such as improvement of food quality which makes them beneficial over chemical fertilizers^17,18^.

One of the most important components of organic fertilizers is the microbial biomass held together in a partially-degraded matrix between soil particles, beside the inorganic component, and this makes them suitable candidates for soil conditioning^92^. Globally, the over-dependence on fertilizers from chemical origin has maximally reduced the quality of soil, increased toxicity to soil beneficial microbes, and promoted freshwater pollution by heavy metals and other chemicals^13^. Therefore, organic fertilizers are vital in providing numerous sustainable benefits which include the enhancement of the quality of soil and the resulting products besides the provision of human and animal well-being^17,18^.

### Field assessments

From the field experiments conducted, experiments involving papaya fruit peel organic fertilizer performed better than the controls as shown in the performance of the maize plants measured throughout the experimental period i.e., 15 to 75 DaS especially at mid to high rates of organic fertilizer application i.e., 30 to 60 kg N/ha. Among all parameters evaluated, the inorganic fertilizer only recorded better results in the values obtained for the length of the root while the organic fertilizers outperformed the inorganic fertilizer in every other parameter. This implies that the organic fertilizer is composed of more nutrients that were readily made available for the plants for performance improvement. Also, the results further show that the nutrients in the organic fertilizer were slowly released to the plant’ roots thereby causing a gradual and steady plant growth as witnessed in the increased values recorded for all the parameters from the time measurement commenced i.e., 15 DaS through to the end of the experiments. Even though, growth was slow at the earlier stage (15 to 30 DaS) and was more conspicuous later on, a trend of steady plant growth was established in this study which further validates the presence and release of plant' beneficial nutrients beside continuous microbial interactions. This assertion supports an earlier study which reported that the nutrient and elemental composition of digestates is usually high since the nutrients originally present in the raw materials used for their production usually remains in them at elevated levels even after digestion thus explaining their huge potentials for replacing fertilizers from a chemical origin in agricultural practices the world over^92^. Babalola^93^ and Suarez et al.^94^ have also affirmed that activities such as plant growth stimulation facilitated by the fixing of atmospheric nitrogen, solubilization, and mobilization of phosphorus, iron sequestration by the actions of siderophores, and production of phytohormones makes organic fertilizers more efficient than inorganic fertilizers. The use of inorganic fertilizers has several negative impacts on the environment besides that they release nutrients to the soil in a non-sustainable manner^55^. All these factors give organic fertilizers an advantage over inorganic fertilizers^58^.

### Improvement of soil quality

One of the major functions of organic fertilizers is soil physical property modification, soil aggregation and hydraulic conductivity improvement, and mechanical resistance reduction^58,95^. Usage as organic fertilizer is a veritable way to efficiently manage anaerobic digestates. This method allows for the maximum recovery of nutrients especially nitrogen and phosphorus besides controlling organic matter loss from soils^83,96^. In this study, soils treated with six organic fertilizer doses were richer in essential nutrients than the controls at the end of the experiments. This is because the papaya fruit peel organic fertilizer is rich in nitrogen and other elements which were slowly released to the soils as a result of lots of microbial interactions especially the actions of siderophores in the rhizosphere and in such a manner as to enrich the soil in the long run. The application of organic fertilizer is currently a popular practice designed for sustainable soil management to improve productivity in agricultural practices^97^. Therefore, the application of digestates organic fertilizers has popularized the utilization of fertilizers in agriculture for the promotion of organic farming and reduced chemical usage globally^58^.

## Conclusion

The application of fertilizers is a common soil management practice that enhances soil fertility and ultimately improves the productivity of agricultural practices. This study has demonstrated that papaya fruit peel is a suitable material for organic fertilizer production via the AD route. The resulting digestate organic fertilizer is rich in both microbes and soil nutrients. When applied to maize plants, the field assessment results were better than those from the NPK 15–15–15 inorganic fertilizer and the control especially at medium to high organic fertilizer application (30 to 60 kg N/ha) rate. The organic fertilizer also enhanced the growth of the crop as well as improved soil fertility. Comparison between the values obtained from the field experiments involving the organic fertilizer and the controls reveals that the organic fertilizer showed better performance in all parameters such as the number of leaves, leaf area, plant height, stem girth, total shoot, and root biomass, and length of the root. However, the chemical fertilizer outperformed all the organic fertilizer applied rates in the average highest size of the corn ear by 1.4%. After harvesting, all the experimental soils recorded an increase in values of all nutrient elements over the control with the highest values recorded in the experiment with 60 kg N/ha. In the 60 kg N/ha soil, nitrogen, phosphorus, and potassium increased by 28, 40, and 22% respectively over the inorganic fertilizer applied experiment while different levels of increases were also recorded for other elements in all experiments. Thus, organic fertilizer produced from papaya fruit peel is a rich source of crop plant’ nutrient and soil beneficial microbes which are needed to maintain soil balance, enhance plant’ growth and wellbeing, increase food production and ultimately ensure food security. Therefore, the use of organic fertilizer is a medium for promoting organic agriculture and a veritable way to overcome the challenges posed by inorganic fertilizers' high rate.

## Supplementary Information

Supplementary Information
