# Supplementary material for: Crop performance and soil fertility improvement using organic fertilizer produced from valorization of Carica papaya fruit peel
Source: Sci Rep. 2021 Feb 25;11:4696. doi: 10.1038/s41598-021-84206-9 (PMC7907234; doi:10.1038/s41598-021-84206-9)
Supplement: Supplementary file 1 — Supplementary Information [file 41598_2021_84206_MOESM1_ESM.docx]

**Crop performance and soil fertility improvement using organic fertilizer produced from valorization of *Carica papaya* fruit peel**

S.O. Dahunsi^1*^, S. Oranusi^2^, V.E. Efeovbokhan^3^, A.T. Adesulu-Dahunsi^4^, J.O. Ogunwole^5^

^1^Microbiology Programme, College of Agriculture, Engineering and Science, Bowen University, Iwo, Osun State, Nigeria

^2^Department of Biological Sciences, Covenant University, Ota, Ogun State, Nigeria

^3^Department of Chemical Engineering, Covenant University, Ota, Ogun State, Nigeria

^4^Food Science and Technology Programme, College of Agriculture, Engineering and Science, Bowen University, Iwo, Osun State, Nigeria

^5^Agriculture Programme, College of Agriculture, Engineering and Science, Bowen University, Iwo, Osun State, Nigeria

Corresponding address: [olatunde.dahunsi@bowen.edu.ng](mailto:olatunde.dahunsi@bowen.edu.ng)

**Table S1: Maize characters and methods of measurement**

| **S/N** | **Plant Character** | **Method of measurement** |
| --- | --- | --- |
| 1 | Leaf number | Counting |
| 2 | Leaf area | Leaf area meter |
| 3 | Plant height | Meter rule (cm) |
| 4 | Stem girth | Veneer Caliper (mm) |
| 5 | Biomass above soil level (fresh) | Weighing scale (g) |
| 6 | Whole root biomass | Weighing scale (g) |
| 7 | Fruit biomass | Weighing scale (g) |
| 8 | Root length | Meter rule (cm) |
| 9 | Whole ear biomass (fresh) | Weighing scale (g) |

**Table S2: Composition of NPK 15-15-15 inorganic fertilizer used in the study**

| **S/N** | **Parameter** | **Value (%)** |
| --- | --- | --- |
| 1. | Nitrogen **(%)** | 15 |
| 2. | P_2_O_3_ **(%)** | 15 |
| 3. | Soluble P_2_O_3_ **(%)** | 13 |
| 4. | K_2_O **(%)** | 15 |
| 5. | Moisture **(%)** | 2 |
| 6.  7. | Particle size (mm)  pH | 1-4.75  7.1±0.12 |


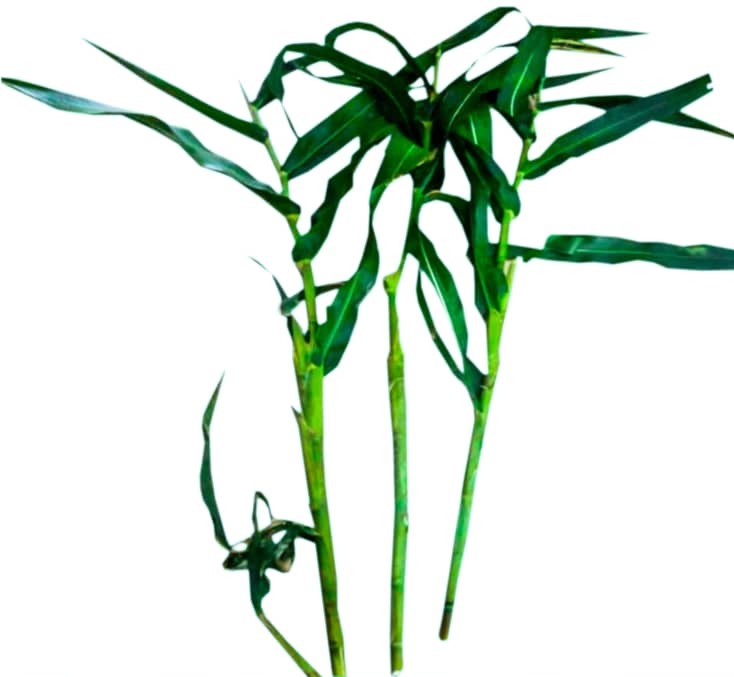


**Figure S1: Complete shoot system from the control (No fertilizer application) and NPK 15-15-15 fertilized experiments**

**
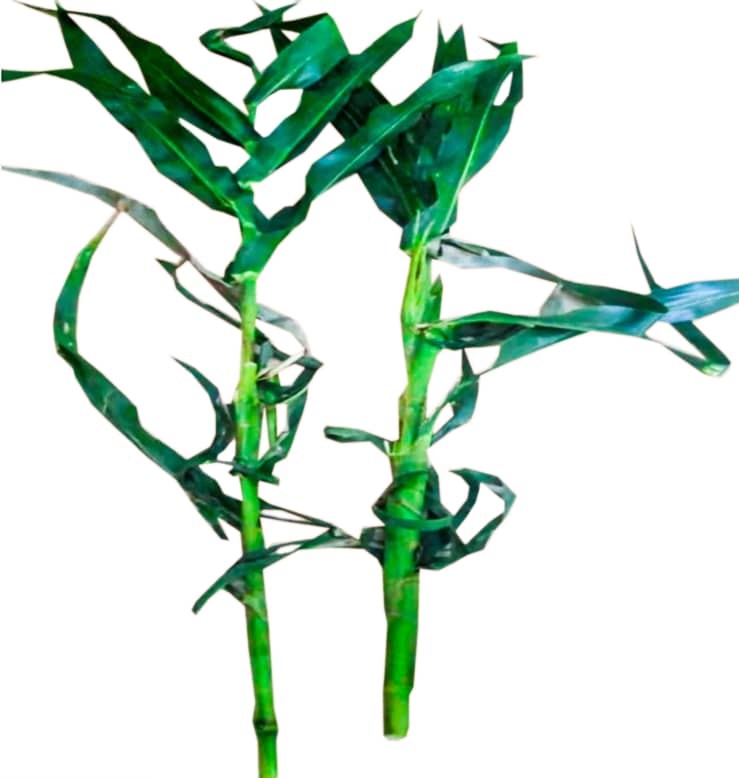
Figure S2: Complete shoot system from the organic fertilized experiments (30 kg N/ha)**
